# Editorial: Dance, embodied agency and neuroplasticity in aging

**DOI:** 10.3389/fnagi.2024.1508074

**Published:** 2024-10-31

**Authors:** Glenna B. Batson, Bettina E. Bläsing, Joseph F. X. DeSouza, Aline Nogueira Haas, Christina E. Hugenschmidt

**Affiliations:** ^1^Physical Therapy, Winston-Salem State University, Winston-Salem, NC, United States; ^2^Department of Sport Science, Bielefeld University, Bielefeld, Germany; ^3^Department of Psychology, Centre for Vision Research, York University, Toronto, ON, Canada; ^4^Department of Physical Education, Physiotherapy and Dance, Federal University of Rio Grande do Sul, Porto Alegre, Brazil; ^5^Section on Gerontology and Geriatric Medicine, Wake Forest University School of Medicine, Winston-Salem, NC, United States

**Keywords:** dance, embodiment, neuroplasticity, aging, cognition

Dance is a multi-modal artistic engagement whose group-delivered protocols have resulted in positive impacts on elderly health (Roberts et al., 2017; Liu et al., 2021). Since the 2022 Research Topic on dance and the elderly was published (Markula et al., 2022), reports from arts-science research collaborations have shown numerous ways person- and community-centered arts-based approaches have extended health across quality-of-life domains among aging adults (Fancourt and Steptoe, 2019; Golden et al., 2023). For dance, such improvements have been reported from training in many different dance styles—structured (modern dance), improvisational, culturally stylized and technologically-assisted. The research is promising, particularly as many barriers to dancing have been removed and ease of access has improved for diverse populations. Nonetheless, important research gaps remain, specifically in articulating the social benefits of dance and their impact on agency (Kaczmarska, 2023; Jensen et al., 2024; Kontos and Grigorovich, 2018). As an aesthetic art form, dance participation couples brain-body health with a range of communicative abilities bearing on relationality and meaning (Warburton, 2011). Data on psychosocial skills of attention, listening, cooperation, self-regulation and empathy are not commonly collected or reported in quantitative research. Valuing such nontangible factors is particularly relevant first in promoting independence and in decreasing the perceived and actual burden of aging on general health and wellbeing. Further, critical to research advancement is the need to distinguish and differentiate functional and neuroplastic outcomes comparing dance protocols with comparable dosages of repetitive fitness exercise (Rehfeld et al., 2018; Müller et al., 2017). Last, the impact of aesthetic factors on health remains understudied (Chappell et al., 2021; Fontanesi and DeSouza, 2021).

For this special Research Topic on dance and health in older adults, our interest focused around the interaction of qualitative and quantitative factors. Specifically, we solicited studies that address how dance participation could foster embodied agency, as well as induce positive neuroplastic changes in the brains of elder adults across different populations.

Manuscripts submitted reflected a global scope of collaborative research between dance educators and neurological and behavioral scientists, including Brazil, Canada, Italy, the United Kingdom, and the United States). Included in this Research Topic are original clinical research, case series, conceptual analysis, and perspectives articles, in which researchers critically analyze interrelated connections and interactions of physical psychological, aesthetic, cultural and social meanings of dance for older persons. Although the Research Topic offers a small sampling of the scope of the topic of embodied agency through dance (nine articles, plus Editorial), they represent an evocative variety of mixed methodologies in both quantitative science and qualitative phenomenological, sociological and cultural research.

The Research Topic opens with a perspective by Sheets-Johnstone, dance philosopher, who emphasizes the need to explicate the neurological and kinesthetic coordination dynamics embodied within dance, dynamics which are critical to engendering health of the whole body.

In a conceptual analysis of an Italian study on dance and Parkinson's disease, Houston extrapolates “soft skills”—”anoetic knowledge,” data which, when expressed through dance, preface a sensate, emotional and affective state of mind—one critical to self- and other-care, vulnerability, patience, and other indicators of meaningful social engagement.

Other samples from the Research Topic include original clinical research: a randomized controlled study on social determinants of health by Worthen-Chaudhuri et al., reporting improvements in autonomy, competence and relatedness for persons with chemotherapy-induced neuropathy. In three studies, researchers designed digitally assisted technology in their dance/movement protocols: First, Delabary et al. reported sustained improvements from Brazilian dance for older adults living with Parkinson's disease, despite switching from on-site to online learning during the COVID 19 epidemic. Second, emplying a protocol or virtual ballet and wellness classes, Harrison et al. reported combined qualitative and quantitaqtive improvements in measures of social efficacy and gait and balance for a group of elderly women. Third, the use of group-delivery of visual assisted feedback of hand gestures by Hansen et al. proved aesthetically “irresistible,” in stimulating movement learning.

The article by Barnstaple et al., speaks to the need for transdisciplinary researchers to rise to the challenge of capturing nuance in “full bodied reporting.” They pose methodological questions and offer guidelines that point the way toward improving sensitivity, reliability and replicability in research designs.

We invite readers to explore all the articles in this Research Topic and thank *Frontiers* for affording us the opportunity to pursue this project. We trust that in reading the articles in this Research Topic, readers will broaden their understanding of ways that dance offers a lived experience of embodiment, relationality, and meaning, integral to building a paradigm of mind and body holism and unity. As scientists consider the value of dance on health and wellbeing in aging, the collective contributions of the artists, participants, and clinicians will assist in building authentic and mutually beneficial relationships among the medical and public communities served.
